# Early Soluble B-Cell Maturation Antigen (BCMA/TNFRSF17) Kinetics as a Molecular Biomarker of Treatment Response in Multiple Myeloma Patients

**DOI:** 10.3390/ijms27125286

**Published:** 2026-06-11

**Authors:** Laura Caponi, Maria Livia Del Giudice, Silvia Ursino, Alice Botti, Alberto Gennari, Aldo Paolicchi, Riccardo Morganti, Gabriele Buda

**Affiliations:** 1 Department of Translational Research and New Technologies in Medicine, University of Pisa, 56126 Pisa, Italy; silvia.ursino@unipi.it (S.U.); alice.botti@med.unipi.it (A.B.); alberto.gennari@ao-pisa.toscana.it (A.G.); aldo.paolicchi@unipi.it (A.P.); 2 Clinical Pathology Laboratory, Pisa University Hospital, 56126 Pisa, Italy; 3 Hematology Unit, Pisa University Hospital, 56126 Pisa, Italy; marialivia.delgiudice@ao-pisa.toscana.it (M.L.D.G.); gabriele.buda@unipi.it (G.B.); 4 Statistical Section, Pisa University Hospital, 56126 Pisa, Italy; r.morganti@ao-pisa.toscana.it; 5 Department of Clinical and Experimental Medicine, University of Pisa, 56126 Pisa, Italy

**Keywords:** soluble B-cell maturation antigen, free light chains, multiple myeloma

## Abstract

Soluble B-cell maturation antigen (sBCMA), generated by shedding of the plasma-cell receptor BCMA/TNFRSF17, is a circulating marker of plasma-cell burden in multiple myeloma (MM). We investigated whether early sBCMA kinetics capture treatment-induced changes in disease biology and predict subsequent Quality of Response (QoR) beyond free light chain (FLC)-based measures. In this prospective longitudinal study, 100 patients with newly diagnosed or relapsed MM starting treatment were evaluated at baseline, 1 month, and 6 months. sBCMA, involved FLC (iFLC), and involved-to-uninvolved FLC ratio (rFLC) were measured, and a 6-month response was assigned according to International Myeloma Working Group criteria. All biomarkers decreased significantly after treatment initiation (*p* < 0.0001). Across disease-status cohorts, sBCMA, but not iFLC or rFLC, differed at baseline and showed significantly different 1-month percentage reductions. Larger early decreases in sBCMA, iFLC, and rFLC were associated with deeper 6-month responses. In ordinal logistic regression including the three biomarkers dichotomized by a 50% reduction threshold at 1 month, only sBCMA remained independently associated with QoR; patients with <50% sBCMA reduction had higher odds of worse 6-month response (OR 5.44, 95% CI 1.58–18.76; *p* = 0.007). These findings support early sBCMA kinetics as a biologically informative marker for short-term response monitoring in MM.

## 1. Introduction

Multiple myeloma (MM) is sustained by clonal plasma-cells whose expansion and persistence are shaped by both tumor-intrinsic programs and bone marrow microenvironmental signals [1]. Among plasma-cell surface receptors, B-cell maturation antigen (BCMA), encoded by *TNFRSF17*, has particular relevance because it is preferentially expressed during terminal B-cell differentiation and is highly relevant to malignant plasma-cell biology [2,3]. In MM models, activation of the APRIL/BAFF-BCMA axis promotes survival and proliferative programs, engaging TRAF-dependent downstream signaling pathways that include NF-kB, MAPK/AKT, JNK and p38-related cascades [4,5]. These molecular features have made BCMA a central therapeutic target and a biologically plausible biomarker in contemporary MM [3].

BCMA is not only a therapeutic antigen but also a measurable circulating biomarker. Membrane-bound BCMA can be cleaved from plasma-cells through gamma-secretase-mediated shedding, generating soluble BCMA (sBCMA), which can be quantified in blood [2,6]. Therefore, serial sBCMA measurements may integrate information about malignant plasma-cell burden, receptor expression/shedding, and treatment-induced changes in the plasma-cell compartment [2,7,8,9]. This molecular rationale distinguishes sBCMA from conventional secretory markers, and supports its evaluation as a dynamic biomarker of early treatment response.

Current MM monitoring relies heavily on serum M-protein and free light chain (FLC) measurements, which are embedded in International Myeloma Working Group (IMWG) diagnostic and response frameworks [10,11,12]. FLC testing is indispensable in many clinical contexts, but interpretation may be affected by renal function, clonal heterogeneity, molecular conformation of light chains, and assay-dependent antibody recognition [10,13,14]. These features can weaken the short-term relationship between measured FLC changes and true tumor-cell reduction, particularly in heterogeneous real-world cohorts.

Although prior studies have associated sBCMA concentrations with tumor burden, disease status, progression risk, treatment response, and survival outcomes [7,8,9,15,16,17], the relative value of early sBCMA kinetics compared with FLC-based measures for predicting response depth remains incompletely defined. We therefore prospectively compared sBCMA, involved FLC (iFLC), and the involved-to-uninvolved FLC ratio (rFLC) in newly diagnosed and relapsed MM patients initiating a new treatment, with the objective of determining whether biomarker changes after one month predict quality of response at six months [11,12].

## 2. Results

During the study period, 100 patients with complete longitudinal data (T0, T1, and T2) were included in the analysis: 40 with newly diagnosed multiple myeloma and 60 with relapsed disease. At the 6-month assessment (T2), responses were complete response (CR), *n* = 6; very good partial response (VGPR), *n* = 34; partial response (PR), *n* = 42; and progressive disease (PD), *n* = 18. In the same period, an additional 37 patients were assessed but were not included because they did not complete the 6-month follow-up (death, *n* = 11; lost to follow-up, *n* = 26).

Baseline demographic and clinical characteristics and response categories of the analyzed cohort are summarized in Appendix A (Table A1).

### 2.1. Baseline Results at Enrollment (T0)

At baseline (T0), median (interquartile range [IQR]) concentrations in the analyzed cohort (*n* = 100) were 161.8 ng/mL (48.24–344.0) for sBCMA, 23.25 mg/L (30.1–1178.0) for iFLC, and 75.11 (7.54–282.2) for the rFLC.

**Figure 1:**
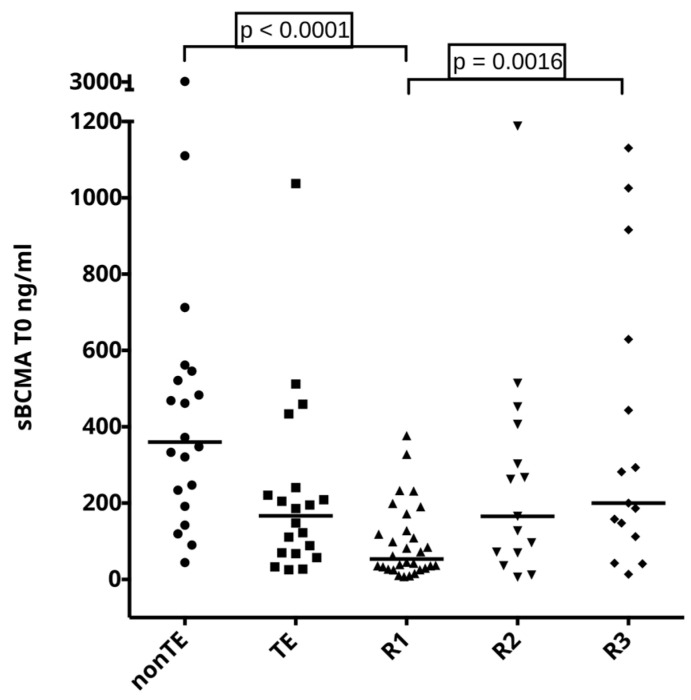
Baseline sBCMA concentrations across the five predefined cohorts. Horizontal bars indicate medians. The overall among-group difference was significant (Kruskal–Wallis, *p* < 0.0001). Significant post hoc pairwise comparisons are annotated on the plot (nonTE vs. R1, *p* < 0.0001; R1 vs. R3, *p* = 0.0016). Abbreviations: nonTE not Transplant Eligible; TE Transplant Eligible; R1 first Relapse; R2 second Relapse; R3 third or more Relapse.

Across the five predefined cohorts (nonTE, TE, R1, R2, and R3), baseline sBCMA differed significantly (Kruskal–Wallis, *p* < 0.0001). Median (IQR) sBCMA was lowest in R1 [53.48 ng/mL (29.03–139.0)] and highest in nonTE [360.0 ng/mL (202.2–539.9)], whereas intermediate values were observed in TE [166.8 ng/mL (68.39–235.7)], R2 [165.3 ng/mL (69.59–406.5)], and R3 [200.2 ng/mL (112.1–629.4)]. Post hoc testing confirmed significant pairwise differences between R1 and nonTE and between R1 and R3 (Figure 1).

By contrast, baseline rFLC did not differ across cohorts (*p* > 0.05).

### 2.2. Trends in Biomarker Concentrations at T1 and T2

In the overall analyzed cohort (*n* = 100), all three biomarkers—sBCMA, iFLC, and the rFLC—decreased from baseline at both 1 month (T1) and 6 months (T2) (Wilcoxon signed-rank test, *p* < 0.0001 for each biomarker for T1 vs. T0 and T2 vs. T0).

**Figure 2:**
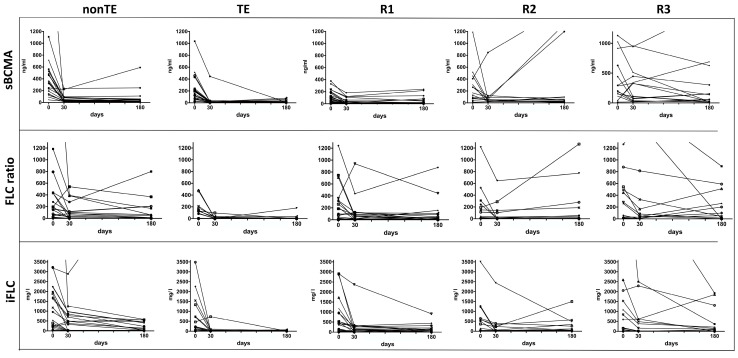
Absolute concentrations of sBCMA, rFLC, and iFLC at T0, T1 and T2 in the five predefined patient groups. Each line from T0 to T1 and then to T2 corresponds to a patient in the respective group. See text for explanation on scales.

Absolute concentrations of sBCMA, rFLC, and iFLC at T0, T1, and T2 in the 5 patient groups are shown in Figure 2. For each biomarker, a scale appropriate for all groups was selected; however, because the concentration range was broad, some data points lying outside the displayed range were not shown.

In subgroup analyses, newly diagnosed patients (nonTE and TE) and patients at first relapse (R1) showed similar downward trajectories for all biomarkers. In contrast, among patients with multiple prior relapses (R2–R3), iFLC decreased over time, whereas changes in rFLC and, particularly, sBCMA were not statistically significant (see Appendix A, Table A2).

### 2.3. Percent Change at 1 Month (T1 vs. T0) and 6 Months (T2 vs. T0)

Because biomarker concentrations spanned a wide range across patients, we additionally analyzed within-patient relative changes. For each patient, the percent change from baseline was calculated for sBCMA, iFLC, and rFLC at 1 month (T1 vs. T0) and 6 months (T2 vs. T0) after treatment initiation. For sBCMA, iFLC, and rFLC, within-patient percent change from baseline was computed at T1 and T2 using: percent change = [(T0 − Tx)/T0] × 100. Negative values indicate biomarker increase.

**Figure 3:**
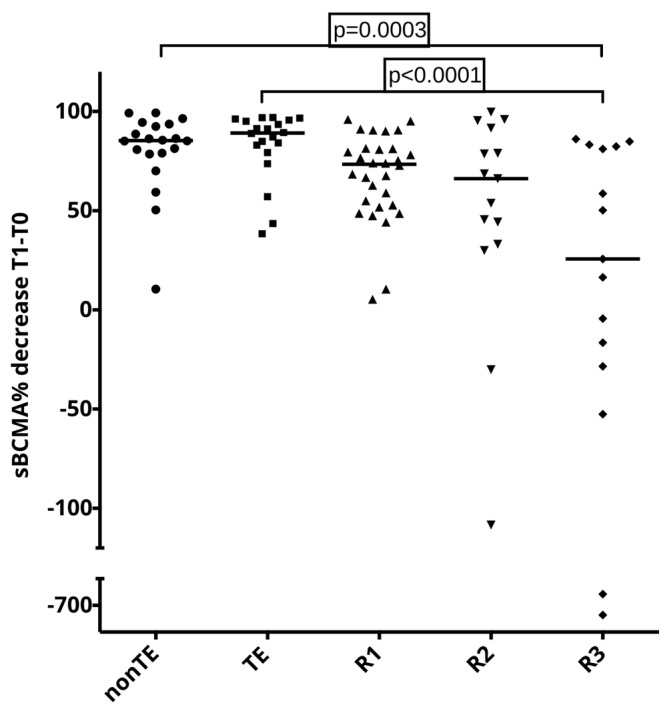
Percent change in sBCMA from baseline to 1 month (T1 vs. T0) across the five predefined cohorts. Percent change differed significantly across cohorts (*p* = 0.0001). Post hoc analysis showed smaller sBCMA changes in R3 than in the newly diagnosed cohorts. Abbreviations: nonTE not Transplant Eligible; TE Transplant Eligible; R1 first Relapse; R2 second Relapse; R3 third or more Relapse.

When comparing within-patient percent changes in sBCMA across the five cohorts (nonTE, TE, R1, R2, and R3), a significant difference was observed at 1 month (T1 vs. T0) (Kruskal–Wallis, *p* = 0.0001). Median (IQR) percent change was lowest in R3 [25.66% (−28.46% to 82.38%)] and highest in the newly diagnosed cohorts [85.36% (78.67–93.36%) in nonTE and 89.14% (80.26–95.56%) in TE], with intermediate values in R1 [73.38% (52.59–81.29%)] and R2 [66.15% (33.07–91.66%)]. Post hoc testing confirmed smaller sBCMA changes in R3 than in nonTE and TE (Figure 3).

In contrast, percent changes in iFLC and rFLC did not differ across cohorts (*p* > 0.05).

At 6 months (T2 vs. T0), within-patient percent changes in sBCMA, iFLC, and rFLC did not differ significantly across the five cohorts (nonTE, TE, R1, R2, and R3) (all *p* > 0.05).

**Figure 4:**
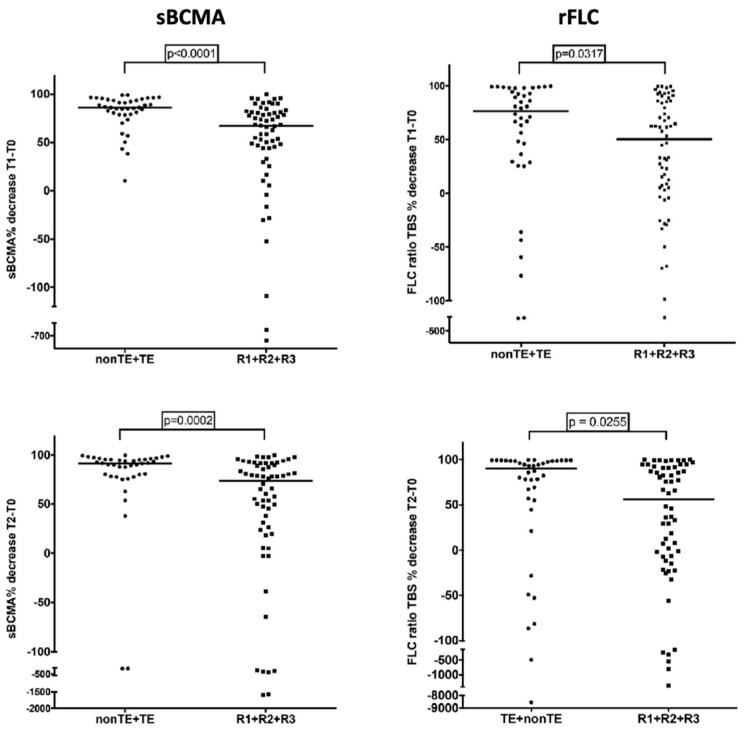
Percent change from baseline for sBCMA and rFLC at 1 month and 6 months. **Upper** panels: percent change at 1 month (T1 vs. T0). **Lower** panels: percent change at 6 months (T2 vs. T0). **Left** panels: sBCMA. **Right** panels: rFLC. Abbreviations: nonTE not Transplant Eligible; TE Transplant Eligible; R1 first Relapse; R2 second Relapse; R3 third or more Relapse.

When cohorts were collapsed into newly diagnosed (nonTE + TE) and relapsed (R1 + R2 + R3) disease, newly diagnosed patients showed larger within-patient percent changes in both sBCMA and rFLC at 1 month and 6 months (Figure 3). For sBCMA, median (IQR) percent change was 86.31% (79.09–94.30%) in newly diagnosed patients versus 67.21% (44.25–81.39%) in relapsed patients at T1 (*p* < 0.0001), and 91.51% (78.63–95.40%) versus 73.48% (24.46–91.19%) at T2 (*p* = 0.0002). For rFLC, the corresponding values were 76.55% (31.19–97.05%) versus 50.24% (5.21–85.68%) at T1 (*p* = 0.0317) and 90.21% (55.56–98.61%) versus 55.94% (−7.22–91.52%) at T2 (*p* = 0.0255) (Figure 4).

### 2.4. Biomarker Percent Changes by Quality of Response (QoR)

Because few patients achieved complete response (CR; *n* = 6), CR and VGPR were combined for response-stratified analyses. We compared within-patient percent changes in sBCMA, rFLC, and iFLC at 1 month (T1 vs. T0) and 6 months (T2 vs. T0) across response categories defined at T2 (CR/VGPR, *n* = 40; PR, *n* = 42; PD, *n* = 18). For all 3 biomarkers, larger decreases were associated with better QoR.

At T1, median (IQR) percent change in sBCMA was 83.99% (73.76–94.27%) in patients achieving CR/VGPR, 78.61% (51.45–86.93%) in those achieving PR, and 44.50% (−28.87–81.73%) in those with PD; overall differences were significant (Kruskal–Wallis, *p* = 0.0007), with a significant pairwise difference between CR/VGPR and PD (*p* = 0.0002). Corresponding values for rFLC were 85.14% (36.70–98.20%), 39.92% (4.64–86.91%), and 53.88% (−0.89–70.12%), respectively (*p* = 0.0030), with significant pairwise differences between CR/VGPR and PR (*p* = 0.0036) and between CR/VGPR and PD (*p* = 0.0039). For iFLC, median (IQR) percent change was 89.06% (60.93–97.58%) in CR/VGPR, 65.50% (41.69–83.89%) in PR, and 49.22% (14.44–75.51%) in PD (*p* = 0.0014), with significant pairwise differences between CR/VGPR and PR (*p* = 0.0093) and between CR/VGPR and PD (*p* = 0.0009).

**Figure 5:**
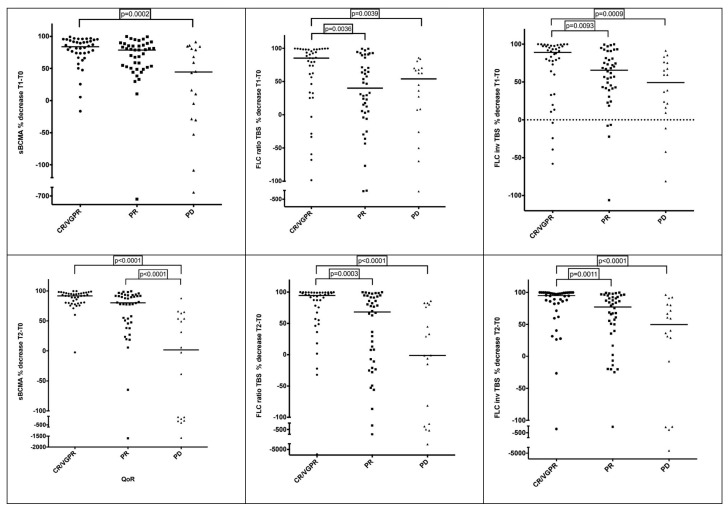
Percent change from baseline in sBCMA, rFLC, and iFLC according to QoR. **Upper** panels show changes at 1 month (T1 vs. T0), **lower** panels show changes at 6 months (T2 vs. T0). Panels are arranged from left to right as sBCMA, rFLC, and iFLC. Abbreviations: CR Complete Response; VGPR Very Good Partial Response; PR Partial Response; PD Progressive Disease.

At T2, differences across QoR categories became more pronounced for all 3 biomarkers (Kruskal–Wallis, *p* < 0.0001 for sBCMA, rFLC, and iFLC). Median (IQR) percent change in sBCMA was 91.90% (80.08–96.28%) in CR/VGPR, 80.27% (47.09–92.08%) in PR, and 1.59% (−235.0% to 56.08%) in PD, with significant pairwise differences between CR/VGPR and PD and between PR and PD (both *p* < 0.0001). For rFLC, the corresponding values were 94.66% (59.57–99.26%), 68.21% (−13.76–93.52%), and −1.22% (−189.6% to 77.30%), with significant pairwise differences between CR/VGPR and PD (*p* < 0.0001) and between CR/VGPR and PR (*p* = 0.0003). For iFLC, median (IQR) percent change was 95.11% (82.37–99.43%) in CR/VGPR, 77.24% (38.47–93.51%) in PR, and 49.74% (−40.30–80.45%) in PD, with significant pairwise differences between CR/VGPR and PD (*p* < 0.0001) and between CR/VGPR and PR (*p* = 0.0011). Significant pairwise comparisons are shown in Figure 5.

Table 1 summarizes the Spearman correlations that reached statistical significance. Notably, the strongest correlations between within-patient percent biomarker changes and QoR, particularly over the T1 vs. T0 interval, were observed in the nonTE group.

### 2.5. Early Prediction of Quality of Response

Given the marked biomarker declines at T1, we evaluated whether early biomarker changes predicted quality of response (QoR) at 6 months (T2) using an ordinal logistic regression model. For each biomarker, the percent change at T1 was dichotomized using an exploratory percentage reduction threshold (≥50% vs. <50%). QoR at T2 was treated as an ordered outcome (CR, VGPR, PR, PD), coded from 1 to 4. In univariable analyses, a ≥50 percentage reduction at T1 in sBCMA, iFLC, and rFLC was associated with a more favorable QoR at T2 (Table 2).

In multivariable ordinal logistic regression using the same ≥50% threshold at T1, only sBCMA remained independently associated with a more favorable QoR at T2. In the ordinal logistic regression model, having a <50 percentage reduction in sBCMA at T1 (vs. ≥50%) was associated with higher odds of worse QoR at T2 (OR, 5.444), corresponding to a 5.44-fold increase in the odds of a 1-category shift toward poorer response (Table 3).

## 3. Discussion

We evaluated the early kinetics of sBCMA and FLC-based measures in patients with newly diagnosed or relapsed MM starting a new line of therapy. Biomarkers were measured at treatment initiation (T0), after 1 month (T1), and after 6 months (T2), and QoR at T2 was assigned according to IMWG criteria. Both sBCMA and FLC-based variables decreased rapidly after treatment initiation, but the principal finding was that only early sBCMA reduction remained independently associated with QoR at six months in multivariable ordinal regression. This supports sBCMA/TNFRSF17 kinetics as a biologically informative, blood-based signal of early treatment response in MM.

From a molecular perspective, this finding is plausible because BCMA/TNFRSF17 is closely linked to the plasma-cell compartment, and its soluble form reflects proteolytic shedding of the receptor into the circulation. Circulating sBCMA should not be interpreted as a simple surrogate for a single molecular process; rather, it may integrate plasma-cell burden, BCMA expression, receptor shedding, treatment-induced cell clearance, and possibly changes in ligand-receptor pathway activity [7]. In this framework, an early fall in sBCMA may capture a biologically meaningful contraction of the plasma-cell compartment before a deeper categorical response is formally assigned by conventional criteria.

A distinctive aspect of this study is the stratification of patients by baseline disease status: newly diagnosed transplant-eligible (TE), newly diagnosed transplant-ineligible (nonTE), and relapsed disease at first, second, or third or later relapse. This design enabled us to examine whether sBCMA and FLC-based measures discriminate across clinically relevant MM subgroups. At baseline (T0), sBCMA—but not iFLC or rFLC—differed across the five cohorts. Specifically, sBCMA was lower in R1 than in R3 and than in newly diagnosed nonTE patients (Figure 1), whereas iFLC and rFLC did not show significant between-cohort differences at T0. These findings suggest that sBCMA may be more sensitive than FLC-based variables to disease-status-related heterogeneity in MM.

The study was originally conceived to evaluate, in parallel, the performance of sBCMA and FLC-based measures as circulating biomarkers of early treatment response in multiple myeloma. In this head-to-head comparison, short-term changes in sBCMA proved more informative than corresponding changes in FLC measures for predicting quality of response at 6 months. When longitudinal changes were considered, sBCMA remained the only biomarker showing differential percentage reductions across cohorts at early follow-up (T1 vs. T0), both in the five-group analysis and after collapsing patients into newly diagnosed versus relapsed disease. sBCMA declines were larger in newly diagnosed patients and smallest in previously treated patients. Under the same regrouping, rFLC—but not iFLC—also showed a between-group difference in percentage reduction, although this pattern was less consistent than that observed for sBCMA (see Figure 3). Consistent with our earlier observations [18], these results further support the usefulness of sBCMA for resolving clinically relevant heterogeneity across MM disease stages.

Because all three biomarkers decreased significantly at T1 in the overall cohort, we examined whether early biomarker changes (T1 vs. T0) predicted QoR at 6 months (T2). Patients who achieved CR/VGPR differed from those with PR or PD in terms of biomarker percentage changes, a separation that was already apparent at T1 and became more pronounced at T2 (Figure 4). This finding indicates that early biomarker kinetics, rather than isolated baseline values, may provide clinically useful information on subsequent response depth. Given that the dynamic variations in the two evaluated biomarkers were expressed as continuous percentages and the clinical outcomes followed a logical ordinal sequence, we were able to move beyond traditional binary metrics, such as sensitivity, specificity, or standard ROC curve analysis. Instead, we implemented an ordinal logistic regression model. This approach allowed us to fully leverage the data from the entire cohort of 100 patients, maintaining statistical robustness regardless of the specific response distribution.

Consequently, to assess the prognostic value of early changes, we analyzed within-patient percentage reduction at T1 using a 50% reduction threshold (≥50% vs. <50%) and modeled QoR as an ordered outcome (CR, VGPR, PR, PD). In univariable analyses, a ≥50% reduction at T1 in sBCMA, iFLC, and rFLC was associated with more favorable QoR at T2. In multivariable ordinal logistic regression, only sBCMA remained independently associated with QoR: patients with a <50% sBCMA reduction at T1 had higher odds of worse response at T2 (OR, 5.44; 95% CI, 1.58–18.76; *p* = 0.007), corresponding to a 1-category shift toward poorer QoR. Thus, early sBCMA kinetics appeared to contain information on subsequent response depth that was not fully captured by iFLC or rFLC.

Several studies have supported sBCMA as a marker of disease burden and outcome in MM, with higher baseline levels associated with shorter progression-free survival and overall survival [7]. More recent work has also emphasized the clinical value of serial sBCMA measurements, showing that the magnitude of sBCMA reduction during therapy is associated with subsequent outcomes, including PFS [8]. Our data extend this literature by focusing on early on-treatment kinetics and by directly comparing sBCMA with FLC-based measures as predictors of response depth. In our cohort, the extent of sBCMA reduction at 1 month, rather than the absolute concentration alone, remained independently associated with QoR at 6 months.

A recent report by Guo et al. [19], analyzing treated MM samples across response categories, suggested that higher sBCMA concentrations are more frequently observed in patients with poorer subsequent responses. While that study emphasized absolute sBCMA levels, our findings indicate that an early relative change—specifically, the percentage decrease shortly after treatment initiation—provides additional prognostic information. This supports the concept that dynamic molecular monitoring of sBCMA may be more informative than a single concentration value for early response assessment.

A key question is why established FLC-based clonality markers, including iFLC and rFLC, did not retain independent predictive value after only 1 month of therapy. This should not be interpreted as lack of clinical value, but rather as a likely consequence of both biological and analytical determinants that may weaken their short-term association with response. Circulating FLC concentrations are influenced by clonal secretion rates, renal clearance, and patient-specific molecular heterogeneity; moreover, the absence of an international reference material limits metrological traceability across assays. Moreover, FLC may exist in different conformational or oligomeric states, altering epitope availability and antibody recognition in immunochemical assays [20], with potential consequences for response categorization and clinical staging [21]. By contrast, sBCMA may provide a more direct circulating readout of BCMA-positive plasma-cell burden, although assay standardization remains a necessary prerequisite before incorporation into routine response-monitoring algorithms.

To make the comparison between biomarkers clinically and analytically meaningful, and in line with how FLC thresholds were historically established, we assessed FLC using the assay configuration most closely aligned with IMWG recommendations—namely Freelite reagents on the Siemens BN II nephelometric platform—which represents a distinctive feature of the present study [12,22]. In principle, the immunochemical issues related to patient-specific analyte heterogeneity and epitope accessibility that affect FLC quantification should be less prominent for sBCMA, which is a single circulating protein target. Nonetheless, recent work has raised concerns about run-to-run consistency and reproducibility for the ELISA format used in many studies, including ours, and discussed possible contributions from long-term storage and protocol-dependent background signal [23]. Importantly, this assay format has underpinned much of the clinical literature on sBCMA in MM, and in our hands, we did not observe excessive background; when repeat testing was required, replicate results were concordant.

Several limitations should be acknowledged. The study was single-center and observational, and treatment regimens were heterogeneous, which reflects real-world practice but limits treatment-specific inference. In addition, only patients with complete 6-month clinical and biospecimen follow-up were included, so survivorship and attrition bias cannot be excluded. The overall sample size was relatively modest (*n* = 100). Nevertheless, although five patient categories were defined at enrollment, they could be collapsed into two sufficiently balanced and clinically coherent groups: newly diagnosed patients (nonTE + TE, *n* = 40) and relapsed patients (R1 + R2 + R3, *n* = 60). This grouping strategy allowed analyses to be extended to broader patient subsets, thereby supporting more robust comparisons at a clinically relevant level.

The CR subgroup was small, requiring combined CR/VGPR analyses for some comparisons. Moreover, no direct comparison with bone marrow MRD, imaging, cell-surface BCMA expression, or gamma-secretase activity was available; therefore, the relationship between circulating sBCMA kinetics, residual disease, and the molecular mechanisms of BCMA shedding could not be directly assessed.

We did not include cytogenetic risk stratification in the analysis, including its most recent update represented by the Consensus Genomic Staging (CGS) framework [24], nor did we account for the specific treatment regimen received by each patient, which may also materially influence biomarker kinetics. Both are important determinants of disease course and will be incorporated in future analyses.

Finally, the 50% one-month sBCMA reduction threshold should be considered exploratory until it is validated in larger independent cohorts and across standardized assay platforms.

In conclusion, early sBCMA/TNFRSF17 kinetics provide a molecularly grounded and clinically accessible signal of treatment response in MM. In this prospective cohort, failure to achieve at least a 50% sBCMA reduction after one month was independently associated with poorer QoR at six months, whereas FLC-based measures did not retain independent predictive value in the multivariable model. These findings support the incorporation of sBCMA kinetics into prospective response-monitoring studies designed to connect circulating molecular signals with conventional response criteria, MRD, and imaging-based disease assessment.

## 4. Materials and Methods

### 4.1. Study Design and Ethics

This single-center, prospective, longitudinal observational study enrolled patients with MM. The study was approved by the local Ethics Committee (Comitato Etico Regionale per la Sperimentazione Clinica della Regione Toscana, approval No. 21711_Buda) and was conducted in accordance with the Declaration of Helsinki. All participants provided written informed consent.

### 4.2. Patients and Study Visits

Consecutive patients with newly diagnosed MM or relapsed MM presenting to the Myeloma Outpatient Clinic, Hematology Unit, Pisa University Hospital, Italy, between 1 March 2022 and 31 December 2024 were eligible.

Clinical and laboratory assessments were performed at treatment initiation (T0), 1 month after treatment initiation ±7 days (T1), and 6 months ±14 days after treatment initiation (T2), according to the routine outpatient follow-up schedule.

Newly diagnosed patients were classified as transplant-eligible (TE) or transplant-ineligible (nonTE) according to standard clinical criteria. Relapsed patients were stratified by relapse number as first relapse (R1), second relapse (R2), or third or later relapse (R3).

To ensure complete longitudinal assessment over 6 months, only patients with available clinical evaluation and biospecimens at T0, T1, and T2 were included; patients who relapsed or died before T2 were excluded.

### 4.3. Sample Collection and Handling

Peripheral blood for sBCMA and FLC testing was collected into lithium-heparin tubes at T0, T1, and T2. Samples were centrifuged at 3000× *g* for 10 min at room temperature and plasma aliquots were immediately stored at −20 °C until analysis. Repeated freeze–thaw cycles were avoided. For each patient, T0, T1, and T2 samples were analyzed within the same analytical run whenever feasible to minimize inter-assay variability.

### 4.4. sBCMA Measurement

sBCMA was measured using the Human BCMA/TNFRSF17 DuoSet ELISA (R&D Systems, Bio-Techne, Minneapolis, MN, USA; cat. n. DY193E) according to the manufacturer’s instructions. Samples and calibrators were assayed in triplicate. Samples were diluted from 1:50 to 1:500 according to the analyte concentration.

### 4.5. Plasma Free Light Chain Measurement

κ and λ FLC concentrations were measured using Freelite^®^ reagents (The Binding Site, Birmingham, UK) on a BN II nephelometer (Siemens Healthineers, Erlangen, Germany) according to the manufacturer’s instructions. Results are reported as mg/L. FLC testing was performed on lithium-heparin plasma after internal verification.

For each patient, the involved FLC (iFLC) was defined as the clonal light chain (κ in κ-restricted disease or λ in λ-restricted disease), and the uninvolved FLC (uFLC) as the opposite light chain. The involved-to-uninvolved FLC ratio (rFLC) was calculated as iFLC/uFLC.

The primary laboratory variables used for analysis were sBCMA, iFLC, and rFLC.

### 4.6. Treatment Response Assessment

Treatment response was assessed at T2 (6 months after treatment initiation) and categorized according to IMWG criteria as complete response (CR), very good partial response (VGPR), partial response (PR), or progressive disease (PD) (11; 12). Response categories were used as the QoR outcome.

### 4.7. Statistical Analysis

Data were analyzed using GraphPad Prism version 6.0 (GraphPad Software, San Diego, CA, USA) and SPSS version 30.0 (IBM Corp., Armonk, NY, USA). Continuous variables are presented as median (interquartile range) and categorical variables as counts (percentages), unless otherwise stated. All tests were two-sided, and *p* < 0.05 was considered statistically significant.

Because biomarker distributions were non-Gaussian, nonparametric methods were used. Comparisons among independent groups were performed using the Kruskal–Wallis test, followed by Dunn’s post hoc test when appropriate. Pairwise comparisons between independent groups were performed using the Mann–Whitney U test. Paired changes over time within individuals were assessed using the Wilcoxon signed-rank test. Associations between biomarkers were evaluated using Spearman’s rank correlation.

Inferential analyses were conducted in the overall cohort and within prespecified subgroups (nonTE, TE, R1, R2, R3). Additional analyses compared newly diagnosed (nonTE + TE) versus relapsed (R1 + R2 + R3) patients. Both pointwise (T0, T1, T2) and longitudinal analyses across the 6-month period were performed to evaluate biomarker trends. To identify factors associated with QoR, univariable analyses were performed after stratifying patients by response category. Given that the dynamic variations in the two evaluated biomarkers were expressed as continuous percentages and the clinical outcomes followed a logical ordinal sequence, we were able to implement a multivariable ordinal logistic regression model with QoR as an ordered outcome; results are reported as odds ratios with 95% confidence intervals.

## Figures and Tables

**Table 1:** Statistically significant Spearman r correlations between within-patient percent biomarker changes and QoR.

**Spearman r correlations T1 vs. T0**			
**Biomarker**	**Overall cohort**	**Significant within the 5 cohorts**	**Significant after regrouping**
sBCMA	r_s_ = 0.3511; *p* < 0.001	nonTE: r_s_ = 0.6850; *p* < 0.001	Newly diagnosed: r_s_ = 0.3976; *p* = 0.011
			Relapsed: r_s_ = 0.3158; *p* = 0.014
rFLC	r_s_ = 0.3090; *p* = 0.002	nonTE: r_s_ = 0.5510; *p* = 0.002	Newly diagnosed: r_s_ = 0.3291; *p* = 0.038
iFLC	r_s_ = 0.3554; *p* < 0.001	nonTE: r_s_ = 0.7294; *p* < 0.001	Newly diagnosed: r_s_ = 0.3413; *p* = 0.031
**Spearman r correlations T2 vs. T0**			
**Biomarker**	**Overall cohort**	**Significant within the 5 cohorts**	**Significant after regrouping**
sBCMA	r_s_ = 0.5548; *p* < 0.001	nonTE: r_s_ = 0.6614; *p* = 0.004	Newly diagnosed: r_s_ = 0.4366; *p* = 0.005
		R1: r_s_ = 0.4716; *p* = 0.008	
		R2: r_s_ = 0.6839; *p* = 0.005	Relapsed: r_s_ = 0.6252; *p* < 0.001
		R3: r_s_ = 0.6452; *p* = 0.011	
rFLC	r_s_ = 0.4695; *p* < 0.001	nonTE: r_s_ = 0.4791; *p* = 0.003	Newly diagnosed: r_s_ = 0.4366; *p* = 0.005
		R1: r_s_ = 0.4463; *p* = 0.013	
		R3: r_s_ = 0.6682; *p* = 0.008	Relapsed: r_s_ = 0.4944; *p* < 0.001
iFLC	r_s_ = 0.4474; *p* < 0.001	nonTE: r_s_ = 0.5413; *p* = 0.014	Newly diagnosed: r_s_ = 0.3817; *p* = 0.015
		R1: r_s_ = 0.4465; *p* = 0.013	Relapsed: r_s_ = 0.4483; *p* < 0.001

**Table 2:** Univariable associations between an early biomarker decrease at T1 (≥50 percentage reduction from baseline, T1 vs. T0) and quality of response (QoR) at 6 months (T2). Biomarkers: sBCMA, rFLC, and iFLC.

Factor	>50% Median (IQR)	≤50% Median (IQR)	*p*-Value
Delta % sBCMA	3 (2–3)	3 (3–4)	<0.001
Delta % rFLC	2 (2–3)	3 (2.5–3)	0.049
Delta % iFLC	3 (2–3)	3 (2–4)	0.034

**Table 3:** Multivariable ordinal logistic regression evaluating the association of a ≥50 percentage reduction at T1 (T1 vs. T0) in sBCMA, rFLC, and iFLC with quality of response (QoR) at T2 (ordered outcome). RC, regression coefficient; OR, odds ratio; CI, confidence interval.

Parameter	RC	OR	95% CI—Inf	95% CI—Sup	*p*-Value
Delta % sBCMA: (0) > 50, (1) ≤ 50	1.694	5.444	1.579	18.764	0.007
Delta % rFLC: (0) > 50, (1) ≤ 50	0.113	1.120	0.305	4.105	0.865
Delta % iFLC: (0) > 50, (1) ≤ 50	−1.113	0.329	0.084	1.286	0.110

## Data Availability

The data presented in this study are available on request from the corresponding author. Biomarkers results are not publicly available due to privacy concerns.

## Abbreviations

The following abbreviations are used in this manuscript:

CR	Complete Response
VGPR	Very Good Partial Response
PR	Partial Response
PD	Progressive Disease
TE	Transplant-Eligible
nonTE	Non-Transplant-Eligible
R1	First Relapse
R2	Second Relapse
R3	Third Or More Relapse
FLC	Free Light Chains
iFLC	Involved FLC
rFLC	Involved/Uninvolved FLC Ratio
sBCMA	Soluble B-Cell Maturation Antigen
MM	Multiple Myeloma

## Appendix A

**Table A1:** Baseline characteristics of the study cohort and quality of response at 6 months. Data are presented as *n* or median (range), as appropriate.

Disease Status	Cohort	Patients, *n*	Age, Years Median (Range)	Sex (F/M)	Involved FLC (Kappa/Lambda)	QoR at 6 Months			
						CR	VGPR	PR	PD
Newly diagnosed	nonTE	20	74 (69–87)	8/12	10/10	1	4	13	2
	TE	20	64 (34–75)	7/13	11/9	1	12	4	3
Relapsed	R1	30	70 (48–80)	12/18	25/5	2	13	13	2
	R2	15	71 (39–80)	4/11	11/4	1	2	8	4
	R3	15	69 (59–86)	6/9	11/4	1	3	4	7
Overall	Total	100	70 (34–87)	37/63	68/32	6	34	42	18

**Table A2:** Statistical significance of longitudinal changes in sBCMA, rFLC, and iFLC by patient subgroup. *p* values for within-group comparisons of biomarker levels at 1 month (T1) and 6 months (T2) versus baseline (T0). *p* values were derived using the Wilcoxon signed-rank test for paired comparisons versus baseline within each subgroup. Two-sided *p* < 0.05 was considered statistically significant.

Group	sBCMA		rFLC		iFLC	
	T1 vs. T0	T2 vs. T0	T1 vs. T0	T2 vs. T0	T1 vs. T0	T2 vs. T0
Overall cohort (*n* = 100)	*p* < 0.0001	*p* < 0.0001	*p* < 0.0001	*p* < 0.0001	*p* < 0.0001	*p* < 0.0001
non-TE	*p* < 0.0001	*p* < 0.0001	*p* = 0.0017	*p* = 0.0083	*p* = 0.0002	*p* = 0.0037
TE	*p* < 0.0001	*p* < 0.0001	*p* < 0.0001	*p* = 0.0009	*p* = 0.0003	*p* < 0.0001
R1	*p* < 0.0001	*p* < 0.0001	*p* = 0.0058	*p* = 0.0030	*p* < 0.0001	*p* < 0.0001
R2	*p* = 0.0151	*p* = 0.2769	*p* = 0.0151	*p* = 0.4212	*p* = 0.0003	*p* = 0.0413
R3	*p* = 0.2524	*p* = 0.1688	*p* = 0.0103	*p* = 0.0554	*p* = 0.0084	*p* = 0.0004
